# Association of Rising Ambient Temperatures with Increased Violence Worldwide: Systematic Review and Meta-Analysis

**DOI:** 10.5811/westjem.42055

**Published:** 2025-09-25

**Authors:** Vivek Chauhan, Suman Thakur, Sagar Galwankar, Sarah Temple

**Affiliations:** * Indira Ghandi Medical College and Hospital, Department of Medicine, Shimla, Himachal Pradesh, India; † Florida State University College of Medicine Emergency Medicine Residency Program at Sarasota Memorial Hospital, Department of Emergency Medicine, Florida

## Abstract

**Introduction:**

Climate change has significantly impacted human health worldwide, contributing to the rise of emerging infectious diseases, allergies, pollution, natural disasters, non-communicable diseases, and malnutrition. One crucial but often overlooked area where climate change has had a notable effect is upon interpersonal violence.

**Methods:**

Following PRISMA guidelines, we searched PubMed and Epistemonikos for studies measuring the effect of temperature on violence. Inclusion criteria encompassed peer-reviewed, English-language articles reporting an association between temperature and violence. Data extraction focused on various forms of violence including homicides, assaults, sexual assaults, suicides, intimate partner violence, riots, and civil wars, and we assessed article quality using Joanna Briggs Institute criteria.

**Results:**

We included a total of 37 studies from 11 countries, three subcontinental regions, and two global-level analyses in this review. Of these, 46% originated from the United States. Rising ambient temperatures were significantly associated with increases in homicides (10 studies), assaults (15 studies), sexual assaults (8 studies), firearm violence (5 studies), intimate partner violence (9 studies), and suicides involving violent methods (9 studies). Conversely, no association was found between temperature and non-violent crimes. Civil wars and riots were also linked to temperature increases in all relevant studies. A meta-analysis of eight studies on violence showed that each 1°C increase in ambient temperature results in 1.64% (95% CI 1.23–2.19%) increase in violence (*P*<.01).

**Conclusion:**

This review demonstrates a significant association between rising temperatures and increased worldwide incidents of violence and self-harm. These findings underscore the urgent need for public health strategies and interventions to mitigate the societal and health impacts of climate change-induced temperature increases.

## INTRODUCTION

The global rise in temperatures due to climate change is a well-documented phenomenon with widespread effects on the environment, economy, and human health. While attention has been focused on the physical and environmental consequences of climate change, less consideration has been given to its impact on social behavior. A recently published overview of 94 systematic reviews published since 2015 on the impact of climate on human health showed that the reviews covered 10 health outcomes with the three most common being infectious diseases, mortality, and respiratory/cardiovascular/neurological diseases.^1^ One critical issue missing from the summary of these 94 systematic reviews was the link between increased temperatures and violence, both interpersonal and collective.

Violence, broadly defined as physical force intended to cause harm, manifests in several forms including homicides, assaults, intimate partner violence (IPV), sexual assaults, suicides, and collective violence such as riots and civil wars. Studies across diverse geographical regions have suggested that warmer temperatures can exacerbate violent behavior, likely due to a combination of physiological and psychological factors. For instance, higher temperatures are thought to increase irritability and aggression, reduce inhibition, and heighten arousal, all of which may contribute to violent acts.^2^ While the relationship between temperature and violence is becoming increasingly recognized, the mechanisms underlying this connection remain poorly understood.

Our goal was to review the existing evidence on the impact of rising temperatures on various forms of violence. Through examining studies from various nations, we sought to provide a comprehensive understanding of this neglected but critical public health issue, highlighting the need for interventions to mitigate violence in a warming world.

## METHODS

We performed a systematic review following the Preferred Reporting Items for Systematic Reviews and Meta-Analyses (PRISMA) methods^3^ using the protocol published in the International Prospective Register of Systematic Review (PROSPERO) (CRD42024581202).

### Search Strategy

We searched two open-access databases, PubMed and Epistemonikos, on July 30, 2024 for peer-reviewed articles on violence and temperature. We operationalized different permutations of each keyword for the two databases as follows:

Pubmed: ((Temperature) OR (‘heat wave’) OR (‘extreme heat’) OR (‘cold wave’) OR (‘extreme cold’)) AND (“Violence” [Mesh])

Epistemonikos: (title:(Violence) OR abstract:(Violence)) AND (title:(Temperature OR “heat wave” OR “cold spell” OR “extreme heat” OR “extreme cold”) OR abstract:(Temperature OR “heat wave” OR “cold spell” OR “extreme heat” OR “extreme cold”))

### Screening and Eligibility

We applied a series of inclusion and exclusion criteria. Articles were included if they were 1) written in English, 2) published in a peer-reviewed journal, and 3) studied the association of temperature with violence. We excluded articles if they 1) were not original, 2) did not describe the effect of temperature on violence, or 3) were a systematic review.

### Extraction and Analysis

Extraction was performed by two investigators independently, who obtained the following information: study characteristics (first author, year of publication, country, years for which violence-related data was collected number of incidents studied); characteristics of violence (assaults, murders, sexual assaults, suicides, robbery, collective violence, civil wars, etc); and outcome data (effect of temperature on violence).

**Population Health Research Capsule**

What do we already know about this issue?

*Research has shown an association between ambient temperatures and interpersonal violence*.

What was the research question?

*What is the impact of rising ambient temperature on various types of violence?*

What was the major finding of the study?

*The meta-analysis of 8 studies on violence showed that each 1°C increase in ambient temperature results in 1.64% (95% CI 1.23–2.19%) increase in violence (P<.01)*.

How does this improve population health?

*Proactive preparedness during extreme heat events could help alleviate the burden of violence-related injuries and mental health issues on public health systems and law enforcement*.

### Study Quality

We used the Joanna Briggs Institute’s critical appraisal checklist for evaluation of the quality of the analytical, cross-sectional studies.^4^ The tool was applied to all studies that were included in the review. None of the studies were excluded based on the outcome of quality assessment. The tool assessed quality using eight questions. A score of 1 was assigned for the answer “Yes,” and a score of 0 was assigned for the answer “No,” “unclear,” or “not applicable.” The scores were graded as low, moderate or high if the total score was ≤ 3, 4–6, and ≥ 7, respectively. The quality assessment was performed independently by two investigators, and any disagreement was settled by discussion.

## RESULTS

### Literature Search

**Figure 1:**
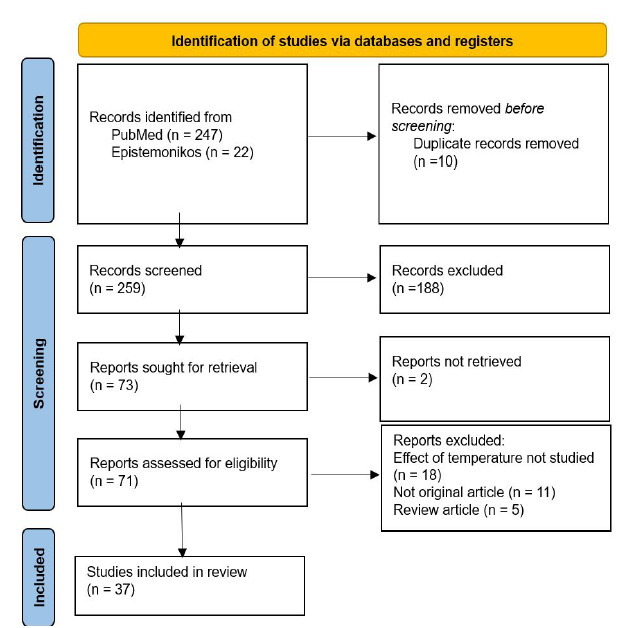
PRISMA flow diagram for the systematic review of association of ambient temperature with violence.

Our initial search resulted in 247 results in PubMed and 22 in Epistemonikos, which were imported into EndNote reference management software (Clarivate, London, United Kingdom). Of these 269 articles, 10 were duplicates, leaving 259 articles for the screening and eligibility stages (Figure 1). Of the 259 articles screened, we excluded 188 that did not meet the inclusion criteria, leaving us with 73 articles for retrieval. We reviewed 71 full texts for eligibility as two could not be retrieved. Of these 71 articles, we excluded five systematic reviews, 11 non-peer reviewed articles, and 18 articles that did not describe the effect of temperature on violence, leaving a total of 37 articles to be included in the final review^5^–^41^ (Table 1). The process of screening and selecting studies is shown in the PRISMA flow diagram (Figure 1).

### Study Characteristics

In our final analysis we included 37 studies from 11 countries (Australia, Belgium, Italy, USA, Switzerland, Hong Kong, South Korea, UK, Taiwan, Spain, and Finland), three subcontinental regions, and two global-level studies (Table 1). Among these, the USA accounted for the largest proportion, with 17 studies (46%), followed by the UK with three studies, and Italy, Australia, and Belgium with two studies each. Interpersonal assaults were the most frequently analyzed dependent variable—examined in 16 studies—followed by suicides involving violent methods in eight studies. Firearm violence, collective violence (riots or civil wars), and intimate partner violence (IPV) were each analyzed in four studies (Table 1). The number of studies exploring the association between temperature and specific forms of violence was as follows: homicides (10 studies); assaults (15 studies); IPV (9 studies); sexual assaults (8 studies); suicides (9 studies), robbery (7 studies); firearm-related violence (5 studies); and civil wars and riots (2 studies each) (Table 2).

Of 37 studies, 28 reported absolute incident counts for various forms of violence (Table 3). The aggregated totals from these studies were as follows:

- **Assaults**: 5,590,523 incidents (across 12 studies)
- **Suicides**: 468,905 incidents (across 7 studies)
- **Collective violence**: 133,500 incidents (across 4 studies)
- **Firearm-related incidents**: 273,377 incidents (across 2 studies)
- **Intimate partner violence**: 164,123 incidents (across 4 studies)

### Definitions

#### Violent Crime

The term *violent crime* encompasses a range of offenses as defined by the authors, including murder, assault, sexual assault, robbery, firearm-related violence, suicides, domestic violence, civil wars, and conflicts.

#### Non-violent and Violent Suicides

Suicides were classified based on *International Classification of Diseases*, 9^th^ Rev (ICD-9) codes. *Non-violent suicides* (ICD-9 codes E950–E952) include those caused by poisoning with solids, liquids, gases, or vapors. All other forms of suicides were categorized as *violent suicides* (ICD-9 codes E953–E959).

#### Temperature

The measurement of temperature variations showed significant heterogeneity in the timeframes used across studies. The timeframes included 17 daily measurements, 10 monthly measurements, three 3- or 6-hourly intervals, three yearly measurements, two measurements at quarterly intervals, one at 6-monthly intervals, and one that recorded weekly measurements (Table 1).

### Outcome Analysis: Effect of Temperature on Violence

All 37 studies used standard statistical methods to assess the relationship between temperature and violence. Of these, 36 studies reported a positive association between rising ambient temperatures and violence, including both self-harm and interpersonal violence. The one study that did not identify a positive association was hospital-based, focusing on assault-related Accident & Emergency Department visits. This study may not accurately reflect the true extent of violence occurring in the broader community.^37^

**Figure 2:**
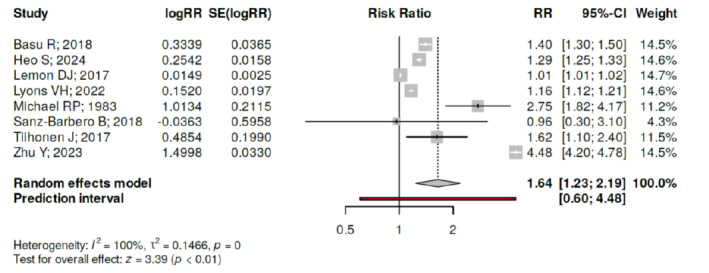
Meta-analysis of eight studies that reported risk of increased violence for each degree Celsius rise in ambient temperature. Based on the investigations performed using a random effects model with inverse variance method, a statistically significant effect can be observed. The summarized effect of each degree Celsius rise of temperature was an increase in violence by 1.64% with a 95% confidence interval of 1.23–2.19. The test for overall effect verified a statistically significant *P* value <.01. A significant heterogeneity was spotted (*P*<.01), suggesting fluctuating effects in extent and/or direction. The I^2^ value shows that 100% of the inconsistency among the cohorts originates from heterogeneity rather than random chance.

The studies summarized their data differently making it difficult for us to pool the data for a meta-analysis. However, eight studies that reported the risk of increase in violence for each degree Celsius rise of ambient temperature were pooled in the meta-analysis (Figure 2).

#### Homicides (Table 3)

The association between temperature and homicides was explored in 10 studies conducted over a cumulative 117 years in four countries: the USA (seven studies), Finland, Australia, and South Korea (one study each).^5^–^7^,^9^,^15^,^23^,^24^,^26^,^38^,^39^ The studies were carried out at the country (five studies), state (one study), and city levels (four studies), with temperature measured in the context of a yearly timeframe in one study, quarterly in one study, monthly in three studies, and daily timeframes in five studies. All 10 studies demonstrated a significant positive association between higher ambient temperatures and increased homicide rates.

#### Assaults (Table 3)

Fifteen studies analyzed the relationship between temperature and assaults over a combined duration of 160 years. These studies spanned eight cities, six countries, one state, and one at a global level.^5^, ^7^, ^13^, ^15^, ^16^, ^18^, ^23^, ^24^, ^26^, ^27^, ^33^, ^34^, ^37^–^39^ Temperature variations were recorded at the following intervals: at 3-hour intervals in one study; daily in seven studies; monthly in four studies; quarterly in two; and yearly in two studies. All studies, except one that focused on a single hospital’s assault-related visits, found a significant association between rising temperatures and increased assaults.^37^

#### Sexual Assaults (Rape) (Table 3)

Eight studies investigated the link between rising temperatures and sexual assaults over a cumulative 100 years, six studies in the USA and two in Australia.^5^–^7^, ^16^, ^23^, ^24^, ^26^, ^38^ Data were collected in three studies at the country level, one at the state level, and four at the city level, with temperature in three studies recorded daily, monthly in two studies, quarterly in two studies, and yearly in one study. All studies identified a significant increase in sexual assaults with higher ambient temperatures.

#### Firearm Violence (Table 3)

Five studies from the USA analyzed firearm violence over a combined 32 years, using daily temperature measurements.^14^, ^17^, ^21^, ^26^, ^35^ Data were collected in three studies at the country level and two studies at city levels. All studies found a significant positive association between rising daily temperatures and increased firearm violence.

#### Domestic or Intimate Partner Violence (IPV) (Table 3)

Nine studies, spanning 41 years, examined the association between ambient temperatures and IPV.^15^, ^16^, ^25^, ^32^, ^34^, ^36^, ^38^, ^40^, ^41^ Three studies represented the USA, two represented Australia, and one each represented Spain, South Korea, and South Asia), with data collected in four studies at the city level, two at the country level, and one each at regional and state levels. In one study, temperature was recorded at 6-hour intervals, while temperature was recorded in five studies on a daily basis, and one study each recorded temperature at quarterly and monthly intervals. Seven studies provided actual numbers, reporting a total of 1,797,812 incidents. All nine studies showed a significant positive association between temperature and IPV.

#### Suicides (Table 3)

Nine studies conducted over a cumulative 147 years examined the relationship between temperature and suicides in six countries, and one study examined that relationship globally.^8^, ^9^, ^12^, ^19^, ^20^, ^22^, ^30^, ^31^, ^40^ Temperature was measured daily in two studies, weekly in one study, monthly in five studies, and yearly in one study. Seven studies reported a total of 468,905 suicide incidents, categorizing them as violent (ICD-9 codes E953–959) or non-violent (ICD-9 codes E950–952). All studies found a significant association between rising temperatures and suicides, with two reporting an effect on only violent suicides, while one found a link to both types.

#### Civil Wars (Table 3)

Two studies examined temperature and civil wars.^10^, ^29^ One analyzed global incidents over 20 years, with yearly temperature variations, while the other focused on East Africa over 19 years, with 6-monthly temperature data. Both studies reported a significant association between higher temperatures and an increase in civil war incidents.

#### Riots (Table 3)

Two studies explored the relationship between temperature and riots.^11^, ^28^ One study in the USA used four years of daily temperature data, while the other, from Sub-Saharan Africa, spanned 32 years using monthly temperature variations. Both studies showed a significant positive association between high temperatures and riots.

#### Non-Violent Crimes (Table 3)

Non-violent crimes, including robbery, arson, theft, and property crimes, were analyzed by Anderson CA et al between 1971–1995 in the USA.^5^–^7^ Using daily, quarterly, and yearly temperature data, the study found no significant association between ambient temperature and non-violent crimes.

#### Trauma Center Admissions (Table 3)

Four studies found that trauma center admissions following assaults increased with rising temperatures, while one study reported no significant association.^9^, ^13^, ^14^, ^26^, ^37^

#### Psychiatric Illnesses (Table 3)

One study examined the link between temperature and psychiatric illnesses presenting to hospitals.^9^ A 10°F increase in temperature during summer resulted in the following: psychosis, +2.9% (0.7–5.2); neurosis, +5.3% (3.8–7.6); self-injury, +5.8% (4.5–7.1); and inflicted injuries: +7.9% (7.3–8.4). During winter, the same temperature increase led to these results: psychosis, +3.9% (1.6–6.3); neurosis, +6.0% (4.5–7.5); self-injury, +7.2% (5.7–8.8); and inflicted injuries, +10.6% (9.8–11.4). The study found a significant association between ambient temperatures and psychiatric illnesses, with a stronger effect observed in winter.

## DISCUSSION

Rising global temperatures, along with other effects of climate change, have shown a clear and significant impact on human health. Various issues such as vector-borne and infectious diseases, environmental pollution hazards, injuries due to natural disasters, allergies, respiratory illnesses, malnutrition, and the mental health impacts of disasters are well-documented concerns.^42^ However, one critical health issue often overlooked is the relationship between global warming and violence. This systematic review highlights the significant influence of rising temperatures on incidents of violence worldwide.

Violence is commonly defined as an act of physical force intended to cause harm and can be broadly categorized into **interpersonal violence** and **group conflicts**. Interpersonal violence includes homicides, assaults, sexual assaults, IPV, and firearm violence, while group conflicts cover riots and civil wars.

Our review demonstrates that rising ambient temperatures are significantly associated with an increase in homicides, assaults, sexual assaults, IPV, and firearm violence. This relationship was observed across various timeframes of temperature measurement—daily, weekly, monthly, quarterly, and yearly—and across a wide range of global communities. Our meta-analysis of eight studies on violence showed that each 1°C increase in ambient temperature results in 1.64% (95% confidence interval 1.23–2.19%) increase in violence (*P*<.01).

Hsiang et al quantified the effect of climate on violence, finding that each one standard deviation increase in temperature results in a 4% increase in interpersonal violence and a 14% increase in intergroup conflict.^43^ Projections suggest that, by the end of the century, climate change could result in an additional 22,000 homicides, 1.2 million aggravated assaults, and 2.3 million simple assaults in the United States alone compared to 2010.^44^ Interestingly, temperature appears to primarily influence violent crimes, such as assaults and homicides, while non-violent crimes, such as robbery, arson, and property crimes, remain unaffected.^5^–^7^ Similarly, studies show a significant rise in violent suicides associated with higher temperatures but no such effect on non-violent suicides, such as those involving poisoning.^9^, ^12^, ^19^, ^20^, ^22^, ^30^, ^31^, ^40^

### The Mechanisms Behind Temperature-Aggression

**The General Aggression Model (GAM)** provides a framework for understanding human responses to provoking situations. It suggests that a person’s reaction—thoughtful or impulsive—depends on their internal state, influenced by affect, cognition, and arousal.^2^ For example:

- **Affect**: Discomfort or pain increases anger and hostility.
- **Cognition**: Media or social priming can condition individuals to be more violent.
- **Arousal**: Situational arousal, such as from exercise, can amplify aggression in response to provocation (a concept explained by the excitation transfer theory).

High temperatures, as per the **temperature-aggression hypothesis**, lead to discomfort that heightens aggression across all three domains of GAM. This increases the likelihood of impulsive violent behaviors, even with minimal provocation.^45^

In regions where warm temperatures are not inherently uncomfortable, the **routine activity theory** offers an alternate explanation. It says that warmer temperatures encourage outdoor activities and social interactions, thereby increasing the likelihood of interpersonal conflicts and exposure to crime-prone environments.^46^

### Implications for Public Health and Safety

Higher ambient temperatures place significant burdens on hospitals and emergency services as they deal with increased cases of heat-related injuries, trauma, assault, mental health crises, and self-harm.^9^, ^13^, ^14^, ^26^, ^31^, ^37^ Targeted police interventions, informed by spatiotemporal analysis, have proven effective in mitigating violence in high-risk areas without displacing the issue to other regions.^13^ Advanced warning systems for heat waves and hotter days can enable hospitals to expect increased arrivals of patients due to violence. However, the extra numbers of arrivals may still be too small to meaningfully impact the needed staffing and resources that should be scheduled to address the increased number of violence-related emergencies that could be anticipated.

### Temperature and Mental Health

While lunar cycles have long been associated with mental health (eg, the term “lunatic”), studies have found no substantial link between lunar phases and psychiatric admissions or discharges.^47^ Conversely, the relationship between temperature and mental health is well-established.^9^ A 10°F (5.6°C) increase in mean apparent temperature during warm seasons correlates with increases in emergency visits for the following: mental health disorders (+4.8%); self-injury/suicide (+5.8%); and intentional injuries/homicides (+7.9%),

**Intimate partner violence** rates are consistently higher on hotter days, even when partner interactions remain constant throughout the year. Zhu et al estimated that a 1°C increase in annual mean temperature results in a 4.49% increase in IPV prevalence.^25^

### Need for Research and Interventions

Despite growing evidence linking temperature to violence, there is limited research on mitigating its effects through heat warning systems and public health strategies. Policymakers and researchers must prioritize developing novel interventions aimed at reducing aggression and violence during hotter days. These could include the following:

- Community-level public health campaigns to “cool tempers” during heatwaves.
- Enhanced preparedness for hospitals and police during extreme weather events.
- Investigation of technologies and policies to create safer environments amid climate change.

As global temperatures continue to rise, addressing this under-recognized public health concern is crucial to protecting communities and reducing the societal impacts of climate change.

## LIMITATIONS

The included studies vary in terms of locations, timeframes, and methods for measuring temperature and violence, limiting generalizability across different regions and cultures. Many studies may not have controlled for other factors such as socioeconomic status, urbanization, or social tensions, which could influence both temperature sensitivity and violence rates. Most studies are cross-sectional, and the scarcity of longitudinal data makes it difficult to establish causal relationships between temperature increases and violence over time. Most studies come from high-income countries, especially the USA and Europe, with fewer from low- and middle-income regions, which limits the applicability to global populations.

Differences in how violence is defined and measured across studies, including official crime statistics vs hospital admissions, may result in discrepancies and fail to capture all forms of violence. Despite these limitations, the review highlights important trends and calls for more targeted research to better understand the link between temperature and violence.

## CONCLUSION

This systematic review highlights a statistically significant relationship—of a small but detectable effect size—between rising ambient temperature and an increase in violence, including homicides, assaults, sexual assaults, suicides, intimate partner violence, firearm violence, riots, and civil wars. Violent behaviors, both interpersonal and group conflicts, are clearly influenced by ambient temperature increases, while non-violent crimes remain largely unaffected. Our findings emphasize the growing burden on hospitals, law enforcement, and public health systems during heat-related emergencies, including violence-related injuries and mental health crises. Targeted interventions, spatiotemporal analysis of hotspots, and early preparedness during extreme heat events are appropriate mitigation steps. As global temperatures continue to rise, integrating violence prevention into broader climate-adaptation frameworks is an appropriate consideration. Focused research and public health strategies are needed to mitigate the behavioral and societal challenges posed by climate change.

## Figures and Tables

**Table 1:** Characteristics of the included studies in the systematic review of the association between temperature and violence.^5^–^41^

First Author	Sample Period	Years	Region	Temperature Time unit	Spatial unit	Main variable	JBI Score
Anderson CA;1987	1980–82	3	USA	Daily	City	Assault	7
Anderson CA;1984	1971–80	10	USA	Quarterly	Country	Assault	6
Anderson CA;1997	1950–95	45	USA	Yearly	Country	Assault	6
Bar S;2022	1995–2016	22	Switzerland	Daily	Country	Suicides	6
Basu R;2018	2005–13	9	USA	Daily	State	Assault	7
Bolfrass A;2015	1994–2014	21	Global	Yearly	Country	Riot/Civil War	7
Carlsmith JM;1979	1967–71	5	USA	Daily	Country	Riot/Civil War	7
Chau PH;2020	1976–2014	39	HongKong	Monthly	SAR	Suicides	6
Cook MR;2012	2008–09	2	USA	Daily	City	Assault	7
Dana EG;2018	2007–11	5	USA	Daily	County	Firearm violence	6
Heo S;2024	2016–20	5	South Korea	Daily	Country	Assault	5
Hodgkinson T;2023	2008–19	12	Australia	Quarterly	State	Assault	6
Jude Kieltyka;2016	1999–2012	14	USA	Daily	City	Firearm violence	7
Lemon DJ;2017	2014–16	3	UK	Daily	County	Assault	7
Lin HC;2008	1997–2003	7	Taiwan	Monthly	Country	Suicides	7
Linkowsky P;1992	1969–84	16	Belgium	Monthly	Country	Suicides	7
Lyons VH;2022	2015–20	6	USA	Daily	Country	Firearm violence	6
Maes M;1994	1979–87	9	Belgium	Weekly	Country	Suicides	6
Mares D;2013	1990–2009	20	USA	Monthly	City	Assault	7
Michael RP;1983	1981–84	4	USA	Monthly	Country	IPV	6
Michael RP;1986	1975–79	5	USA	Monthly	Country	IPV	7
Michel SJ;2016	2008–13	6	USA	Daily	City	Assault	6
Moore SC;2022	2016–19	4	UK	Daily	City	Assault	7
O’Loughlin J;2014	1990–2009	20	East Africa	6-Monthly	Sub-Continent	Riot/Civil War	6
O’Loughlin J;2012	1980–2012	21	Sub-Saharan Africa	Monthly	Sub-Continent	Riot/Civil War	6
Preti A;1998	1984–95	12	Italy	Monthly	Country	Suicides	6
Preti A;2000	1984–95	12	Italy	Monthly	Country	Suicides	7
Rotton J;1985	1975–76	2	USA	Daily	City	Assault	6
Rotton J;2000	1994–95	2	USA	3-hourly	City	Assault	6
Rotton J;2001	1987–88	2	USA	6-hourly	City	IPV	6
Ruderman D;2021	2014–19	6	USA	Daily	Country	Firearm violence	5
Sanz-Barbero B;2018	2008–16	9	Spain	Daily	City	IPV	5
Sivarajasingam V;2004	1995–2000	6	UK	Monthly	City	Assault	6
Stevens HR;2024	2013–18	6	Australia	Daily	City	Assault	7
Tiihonen J;2017	1996–2013	18	Finland	Monthly	Country	Assault	6
Zhou X;2024	1990–2019	30	Global	Yearly	Global	Suicides	7
Zhu Y;2023	2010–18	9	South Asia	Daily	Sub-Continent	IPV	7

*JBI, J*oanna Briggs Institute quality assessment score; *USA*, United States of America; *UK*, United Kingdom; *SAR*, Special Administrative Region; *IPV*, intimate partner violence.

**Table 2:** Types of violence reported by the studies included in a systematic review of the association between temperature and violence.^5^–^41^

First author	Years	Region	Temperature time unit	Spatial unit	Dependent variables
Anderson CA;1987	3	USA	Daily	City	Homicide, Assault, Rape, Robbery, Theft
Anderson CA;1984	10	USA	Quarterly	Country	Homicide, Rape, Robbery, Theft
Anderson CA;1997	45	USA	Yearly	Country	Homicide, Assault, Rape, Robbery, Property Crime
Bar S;2022	22	Switzerland	Daily	Country	Suicide
Basu R;2018	9	USA	Daily	State	Homicide, Psychosis, Neurosis, Self-injury/Suicide
Bolfrass A;2015	21	Global	Yearly	Country	Civil War
Carlsmith JM;1979	5	USA	Daily	Country	Riot
Chau PH;2020	39	Hong Kong	Monthly	SAR	Suicide (violent and non-violent)
Cook MR;2012	2	USA	Daily	City	Assault
Dana EG;2018	5	USA	Daily	County	Firearm violence
Heo S;2024	5	South Korea	Daily	Country	Homicide, Assault, Robbery, IPV
Hodgkinson T;2023	12	Australia	Quarterly	State	Assault, Rape, Robbery, IPV
Jude Kieltyka;2016	14	USA	Daily	City	Firearm violence
Lemon DJ;2017	3	UK	Daily	County	Assaults
Lin HC;2008	7	Taiwan	Monthly	Country	Suicide (violent and non-violent)
Linkowsky P;1992	16	Belgium	Monthly	Country	Suicide (violent and non-violent)
Lyons VH;2022	6	USA	Daily	Country	Firearm violence
Maes M;1994	9	Belgium	Weekly	Country	Suicide (violent)
Mares D;2013	20	USA	Monthly	City	Homicide, Assault, Rape, Robbery
Michael RP;1983	4	USA	Monthly	Country	Homicide, Assault, Rape, Robbery
Michael RP;1986	5	USA	Monthly	Country	IPV
Michel SJ;2016	6	USA	Daily	City	Homicide, Assault, Rape, Firearm Violence
Moore SC;2022	4	UK	Daily	City	Assault
O’Loughlin J;2014	20	East Africa	6-Monthly	Sub-Continent	Riot
O’Loughlin J;2012	21	Sub-Saharan Africa	Monthly	Sub-Continent	Civil War
Preti A;1998	12	Italy	Monthly	Country	Suicide (violent and non-violent)
Preti A;2000	12	Italy	Monthly	Country	Attempted suicide (violent, non-violent)
Rotton J;1985	2	USA	Daily	City	Assault
Rotton J;2000	2	USA	3-hourly	City	IPV
Rotton J;2001	2	USA	6-hourly	City	Assault, IPV
Ruderman D;2021	6	USA	Daily	Country	Firearm violence
Sanz-Barbero B;2018	9	Spain	Daily	City	IPV
Sivarajasingam V;2004	6	UK	Monthly	City	Assault related A&E admissions
Stevens HR;2024	6	Australia	Daily	City	Homicide, Assault, Rape, IPV
Tiihonen J;2017	18	Finland	Monthly	Country	Homicide, Assault
Zhou X;2024	30	Global	Yearly	Global	Attempted suicide, IPV
Zhu Y;2023	9	South Asia	Daily	Sub-Continent	IPV

*USA*, United States of America; *UK*, United Kingdom; *SAR*, Special Administrative Region; *IPV*, intimate partner violence; *A&E*, Accident and Emergency Department.

**Table 3:** Incidents of violence as reported by the studies included in the systematic review of the association between temperature and violence.

First author	Sample period	Region	Main dependent variable	Incidents
Basu R;2018	2005–13	USA	Assault	1,041,433
Cook MR;2012	2008–09	USA	Assault	414
Heo S;2024	2016–20	South Korea	Assault	414,6474
Hodgkinson T;2023	2008–19	Australia	Assault	4,380
Lemon DJ;2017	2014–16	UK	Assault	4,262
Michel SJ;2016	2008–13	USA	Assault	32,654
Rotton J;1985	1975–76	USA	Assault	18,687
Rotton J;2000	1994–95	USA	Assault	57,834
Sivarajasingam V;2004	1995–2000	UK	Assault	19,264
Stevens HR;2024	2013–18	Australia	Assault	182,962
Tiihonen J;2017	1996–2013	Finland	Assault	55,159
Bolfrass A;2015	1994–2014	Global	Riot/Civil War	44,498
Carlsmith JM;1979	1967–71	USA	Riot/Civil War	102
O’Loughlin J;2014	1990–2009	East Africa	Riot/Civil War	72,541
O’Loughlin J;2012	1980–2012	Sub-Saharan Africa	Riot/Civil War	16,359
Jude Kieltyka;2016	1999–2012	USA	Firearm violence	156,866
Lyons VH;2022	2015–20	USA	Firearm violence	116,511
Rotton J;2001	1987–88	USA	IPV	10,765
Sanz-Barbero B;2018	2008–16	Spain	IPV	38.088
Michael RP;1983	1975–79	USA	IPV	27,000
Zhu Y;2023	2010–18	South Asia	IPV	52,567
Bar S;2022	1995–2016	Switzerland	Suicides	24,067
Chau PH;2020	1976–2014	Hong Kong	Suicides	7,944
Lin HC;2008	1997–2003	Taiwan	Suicides	18,130
Linkowsky P;1992	1969–84	Belgium	Suicides	25,075
Preti A;1998	1984–95	Italy	Suicides	43,755
Preti A;2000	1984–95	Italy	Suicides	27,456

*USA*, United States of America; *UK*, United Kingdom; *IPV*, intimate partner violence.

## References

[1] Rocque RJ, Beaudoin C, Ndjaboue R (2021). Health effects of climate change: an overview of systematic reviews. BMJ open.

[2] Allen JJ, Anderson CA, Bushman BJ (2018). The General Aggression Model. Curr Opin Psychol.

[3] Liberati A, Altman DG, Tetzlaff J (2009). The PRISMA statement for reporting systematic reviews and meta-analyses of studies that evaluate health care interventions: explanation and elaboration. PLoS med.

[4] Munn Z, Moola S, Lisy K (2019). Chapter 5 Systematic Reviews of Prevalence and Incidence.

[5] Anderson CA (1987). Temperature and aggression: effects on quarterly, yearly, and city rates of violent and nonviolent crime. J Pers Soc Psychol.

[6] Anderson CA, Anderson DC (1984). Ambient temperature and violent crime: tests of the linear and curvilinear hypotheses. J Pers Soc Psychol.

[7] Anderson CA, Bushman BJ, Groom RW (1997). Hot years and serious and deadly assault: empirical tests of the heat hypothesis. J Pers Soc Psychol.

[8] Bär S, Bundo M, de Schrijver E (2022). Suicides and ambient temperature in Switzerland: a nationwide time-series analysis. Swiss Med Wkly.

[9] Basu R, Gavin L, Pearson D (2018). Examining the association between apparent temperature and mental health-related emergency room visits in California. Am J Epidemiol.

[10] Bollfrass A, Shaver A (2015). The effects of temperature on political violence: global evidence at the subnational level. PloS one.

[11] Carlsmith JM, Anderson CA (1979). Ambient temperature and the occurrence of collective violence: a new analysis. J Pers Soc Psychol.

[12] Chau PH, Yip PSF, Lau HYE (2020). Hot weather and suicide deaths among older adults in Hong Kong, 1976–2014 a retrospective study. Int J Environ Res Public Health.

[13] Cook MR, Emick D, Foley C (2012). Daily temperature predicts assault and may allow more efficient policing. Am Surg.

[14] Dana EG, Kara ER, Jennifer A (2018). Predictors of firearm violence in urban communities a machine learning approach. Health Place.

[15] Heo S, Choi HM, Lee JT (2024). A nationwide time-series analysis for short-term effects of ambient temperature on violent crime in South Korea. Sci Rep.

[16] Hodgkinson T, Corcoran J, Andresen MA (2023). Violent assault geographies in northeastern Australia. PloS one.

[17] Kieltyka J, Kucybala K, Crandall M (2016). Ecologic factors relating to firearm injuries and gun violence in Chicago. J Forensic Leg Med.

[18] Lemon DJ, Partridge R, Pan-Dorset Cardiff Model team (2017). Is weather related to the number of assaults seen at emergency departments?. Injury.

[19] Lin HC, Chen CS, Xirasagar S (2008). Seasonality and climatic associations with violent and nonviolent suicide: a population-based study. Neuropsychobiology.

[20] Linkowski P, Martin F, De Maertelaer V (1992). Effect of some climatic factors on violent and non-violent suicides in Belgium. J Affect Disord.

[21] Lyons VH, Gause EL, Spangler KR (2022). Analysis of daily ambient temperature and firearm violence in 100 US Cities. JAMA Netw Open.

[22] Maes M, De Meyer F, Thompson P (1994). Synchronized annual rhythms in violent suicide rate, ambient temperature and the light-dark span. Acta psychiatr Scand.

[23] Mares D (2013). Climate change and levels of violence in socially disadvantaged neighborhood groups. J Urban Health.

[24] Michael RP, Zumpe D (1983). Annual rhythms in human violence and sexual aggression in the United States and the role of temperature. Soc Biol.

[25] Michael RP, Zumpe D (1986). An annual rhythm in the battering of women. Am J Psychiatry.

[26] Michel SJ, Wang H, Selvarajah S (2016). Investigating the relationship between weather and violence in Baltimore, Maryland, USA. Injury.

[27] Moore SC, Woolley TE, White J (2022). An exploration of the multiplicative effect of “other people” and other environmental effects on violence in the night-time environment. Int J Environ Res Public Health.

[28] O’Loughlin J, Linke AM, Witmer FD (2014). Effects of temperature and precipitation variability on the risk of violence in Sub-Saharan Africa, 1980–2012. Proc Natl Acad Sci U S A.

[29] O’Loughlin J, Witmer FD, Linke AM (2012). Climate variability and conflict risk in East Africa, 1990–2009. Proc Natl Acad Sci U S A.

[30] Preti A, Miotto P (1998). Seasonality in suicides: the influence of suicide method, gender and age on suicide distribution in Italy. Psychiatry Res.

[31] Preti A, Miotto P (2000). Influence of method on seasonal distribution of attempted suicides in Italy. Neuropsychobiology.

[32] Rotton J, Cohn EG (2001). Temperature, routine activities, and domestic violence: a reanalysis. Violence Vict.

[33] Rotton J, Cohn EG (2000). Violence is a curvilinear function of temperature in Dallas: a replication. J Pers Soc Psychol.

[34] Rotton J, Frey J (1985). Air pollution, weather, and violent crimes: concomitant time-series analysis of archival data. J Pers Soc Psychol.

[35] Ruderman D, Cohn EG (2021). Predictive extrinsic factors in multiple victim shootings. J Prim Prev.

[36] Sanz-Barbero B, Linares C, Vives-Cases C, González JL, López-Ossorio JJ, Díaz J (2018). Heat wave and the risk of intimate partner violence. Sci Total Environ.

[37] Sivarajasingam V, Corcoran J, Jones D (2004). Relations between violence, calendar events and ambient conditions. Injury.

[38] Stevens HR, Graham PL, Beggs PJ (2024). Associations between violent crime inside and outside, air temperature, urban heat island magnitude and urban green space. Int J Biometeorol.

[39] Tiihonen J, Halonen P, Tiihonen L (2017). The association of ambient temperature and violent crime. Sci Rep.

[40] Zhou X, Li R, Cheng P (2024). Global burden of self-harm and interpersonal violence and influencing factors study 1990–2019: analysis of the global burden of disease study. BMC Public Health.

[41] Zhu Y, He C, Bell M (2023). Association of ambient temperature with the prevalence of intimate partner violence among partnered women in low- and middle-income South Asian countries. JAMA Psychiatry.

[42] Rossati A (2017). Global warming and its health impact. Int J Occup Environ Med.

[43] Hsiang SM, Burke M, Miguel E (2013). Quantifying the influence of climate on human conflict. Science.

[44] Ranson M (2014). Crime, weather, and climate change. J Environ Econ Manage.

[45] Anderson CA, Anderson KB, Dorr N (2000). Temperature and aggression. Adv Exp Soc Psychol.

[46] Cohn EG (1990). Weather and crime. Brit J Criminol.

[47] Gupta R, Nolan DR, Bux DA (2019). Is it the moon? Effects of the lunar cycle on psychiatric admissions, discharges and length of stay. Swiss Med Wkly.

